# A requirement for *slc15a4* in imiquimod-induced systemic inflammation and psoriasiform inflammation in mice

**DOI:** 10.1038/s41598-018-32668-9

**Published:** 2018-09-27

**Authors:** Alexis D. Griffith, Asifa K. Zaidi, Ashley Pietro, Matthew Hadiono, Jessica S. Yang, Rachel Davis, Daniel L. Popkin

**Affiliations:** 1 Department of Dermatology, Case Western Reserve University Hospitals, Cleveland, OH 44106 USA; 2 Departments of Dermatology, Pathology, Molecular Biology and Microbiology, Case Western Reserve University Hospitals, Cleveland, OH 44106 USA

## Abstract

There is competing evidence that plasmacytoid dendritic cells (pDC), the most potent source of IFN-I, may initiate psoriasis. We targeted pDC function using the *slc15a4*^*feeble*^ loss-of-function mouse whose pDC are unresponsive to TLR agonists. *slc15a4*^*feeble*^ treated with the topical TLR7-agonist imiquimod (IMQ) demonstrated decreased epidermal thickening 24 hours post-treatment which was more pronounced by day 5 as compared to wildtype mice. These findings were specific to the acute IMQ model and not the protracted IL23 model that drives inflammation downstream of TLR activation. Systemically, *slc15a4* was required for IMQ-induced weight loss and cutaneous accumulation of CD4+ and Siglec H+, but not CD11b+ cells. Consistent with this phenotype and the function of *slc15a4*, induction of IFN-I was virtually absent systemically and via cutaneous gene expression. Induction of other inflammatory cytokines (cytokine storm) was modestly blunted in *slc15a4*^*feeble*^ except for inflammasome-associated genes consistent with *slc15a4* being required for TLR7-mediated (but not inflammasome-mediated) inflammation downstream of IMQ. Surprisingly, only IFN-I gene expression was suppressed within IMQ-treated skin. Other genes including conserved psoriasiform trademark gene expression were augmented in *slc15a4*^*feeble*^ versus littermate controls. Taken together, we have identified a role for *slc15a4* but not canonical psoriasiform genes in the imiquimod model of psoriasiform dermatitis.

## Introduction

Psoriasis is a chronic autoimmune disease affecting approximately 2% of the population^1^. While the cause of psoriasis is unknown, there is competing evidence that plasmacytoid dendritic cells (pDC) may be central to disease development^2–5^. pDCs are responsible for producing ~1000 times more type I interferon (IFN-I) than any other cell type. Thus, despite comprising only ~1% of the already rare dendritic cell population, pDCs account for approximately 50% of all circulating IFN-I following infection^6^. It has been proposed that pDCs in genetically predisposed individuals under certain environmental conditions and through their secretion of IFN-I, activate autoimmune T cells, in turn, eliciting a psoriasiform phenotype^4^.

### Slc15a4

*Solute carrier gene family 15 member 4 (SLC15A4)* is a twelve membrane spanning protein also known as peptide/histidine transporter 1 (PHT1). *Slc15a4* cellular gene expression essentially mirrors TLR7 and 9 expression being most highly expressed in antigen presenting cells, specifically plasmacytoid dendritic cells (pDC) and B cells^7–9^. Consistently, a functional role for *slc15a4* has only been demonstrated in these two cell types to date. *Slc15a4* contains an acidic dileucine motif which likely mediates localization to the endosomal compartment via AP-3^7^. Subsequent biogenesis of lysosome-related organelles (LROs) harboring TLR7 and TLR9 is defective without *slc15a4*. Thus TLR7 and TLR9 sensing is specifically lost in *slc15a4* mutant mice resulting in acute loss of IFN-I production secondary to defective pDC within 6 hours post CpG injection^7^ and loss of B cell functional responses reported as early as 48 hours after TLR7 and TLR9 stimulation^10^.

### slc15a4 phenotypes *in vivo*: SLE, acute and chronic viral infection

Consistent with the specific function and expression of *slc15a4*, *slc15a4*^*feeble*^ loss of function mice are protected in the NZB and Fas(lpr) mouse models of systemic lupus erythematosus (SLE)^11^. GWAS studies have likewise identified *slc15a4* as a genetic locus contributing to human lupus in 6 independent studies^12–17^.

The physiological role of *slc15a4* is likely for protection against pathogens via TLR7/9 sensing. We tested this hypothesis and found that *slc15a4* was not required for control of acute viremia but was necessary for virus-specific T cell activation and chronic viral clearance^18^. Here we extend our analysis of *slc15a4* to examine its role in psoriasiform dermatitis mediated by the canonical TLR7 agonist imiquimod as compared to intradermal IL23 injection.

### Imiquimod (IMQ) model, TLR7, IFN-I and plasmacytoid DC (pDC)

The IMQ model of acute skin inflammation has become the most widely used mouse model in this category over the past several years^19^. TLR7 sensing via pDC is thought to drive massive IFN-I expression within 6 hours that contributes to the induction of multiple inflammatory markers and histologic alterations in the skin seen within 12–24 hours^19^. However, there is also a vehicle-specific TLR7-independent effect which activates the inflammasome and contributes in part to the acanthosis and inflammation present in skin^20,21^. pDC production of IFN-I is an expected dramatic component of the IMQ model and critical to initiate psoriasis in a xenograft model^4^. However, deficiency of the IFN-I receptor was reported to have no effect in two IMQ studies^5,20^, while two subsequent IMQ studies demonstrated a significant role for the IFN-I receptor (IFNAR1)^22,23^. Similarly, pDC which are responsible for ~50% of all acute IFN-I^24^ has been implicated in psoriasis^4^ and the IMQ psoriasiform model. Yet surprisingly, targeting these cells did not result in any significant changes in the IMQ model^5^.

Here, we evaluated the role of *slc15a4* in imiquimod-induced psoriasiform dermatitis. The mouse mutation examined in our study *(feeble)* has a one base-pair mutation in *slc15a4* rendering the transporter inactive but *feeble* mice are otherwise genetically identical to their C57BL/6 J wildtype littermates (WT). Consistently, these mice are indistinguishable from WT during development, including DC and B cell phenotypic populations. However, there is a dramatic loss of DC function in response to TLR stimulation, especially regarding IFN-I secretion^7,10^ as well as a defect in IgG2c class switch recombination during CpG-adjuvanted vaccination^10^. Using this *feeble* model, we found significant decreases in disease initiation and progression in *slc15a4*^*feeble*^ as compared to WT mice both locally and systemically.

## Results

The role of pDC and IFN-I in the IMQ psoriasiform dermatitis model is controversial in addition to the source of IFN-I production^4,5,20,22,23^. Therefore, we examined an alternative target which may also be inhibited therapeutically to treat TLR-pDC driven immune pathology, *slc15a4*. *Slc15a4* is required for pDC TLR7/9 sensing and subsequent TLR driven IFN-I production^7^. We and others found that *slc15a4* was important in disease models^8,11,18,25^. Therefore, we hypothesized that studying *slc15a4* deficiency in the IMQ psoriasiform dermatitis model may clarify the role of the TLR7-pDC-IFN-I pathway of immune pathology. A significant role would also justify *slc15a4* as a specific therapeutic target to alleviate disease.

### slc15a4 ^*feeble*^ mice are more resistant to imiquimod induced weight loss than epidermal skin thickening in an acute psoriasiform dermatitis model, but not resistant to IL23 induced skin inflammation

**Figure 1:**
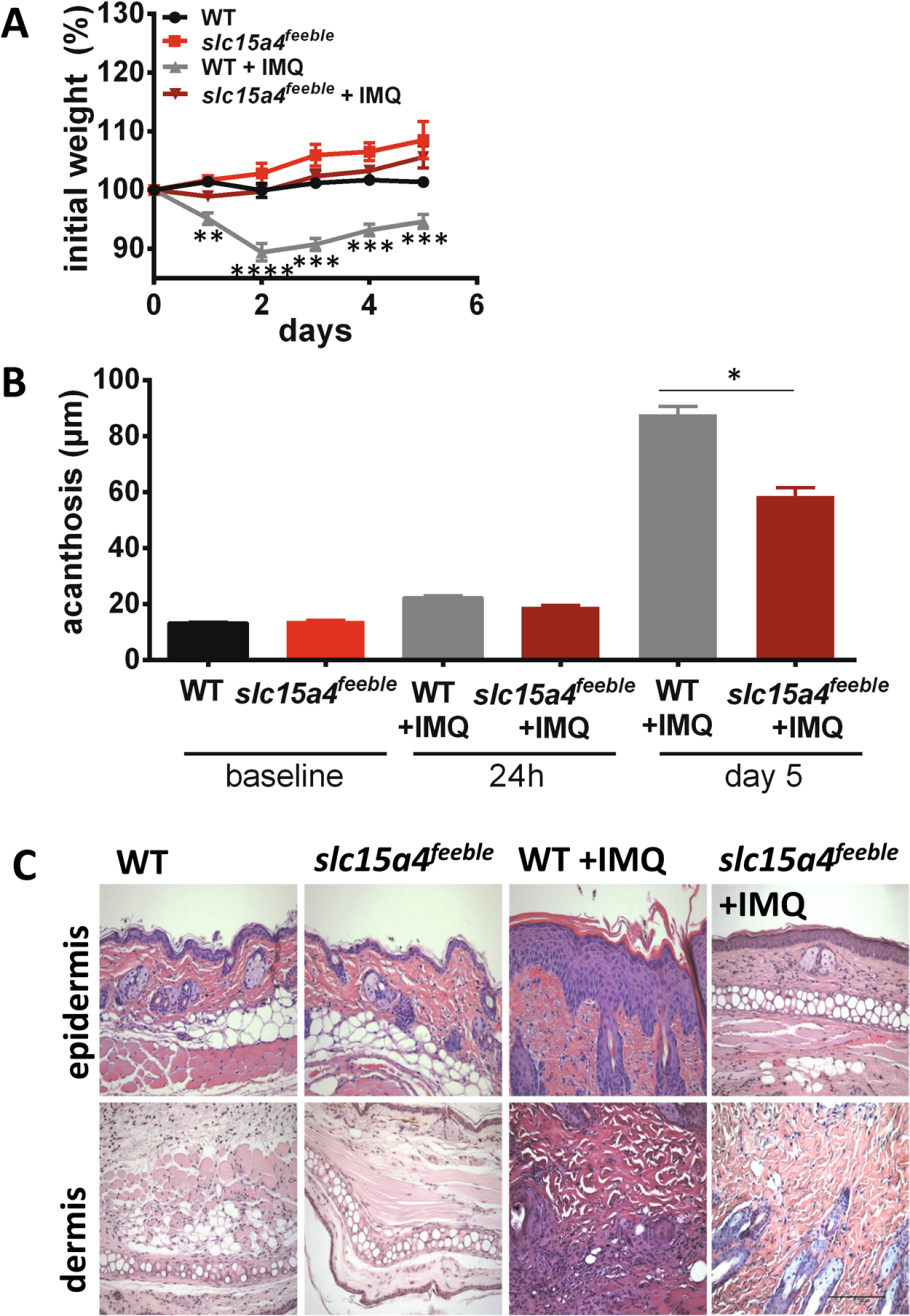
slc15a4 is required for imiquimod induced weight loss and epidermal thickening. *slc15a4*^*feeble*^ and WT mice were treated with imiquimod (IMQ) and euthanized after 24 hours or 5 days. (**A**) Significant IMQ-induced weight loss was observed in WT mice as compared to slc15a4feeble mice (n = 7, 5, 17, 15). (**B**) Attenuated IMQ-induced acanthosis was observed in slc15a4feeble mice at 24 hours and 5 days post daily IMQ treatment compared to WT mice (n = 7, 5, 6, 6, 17, 5). (**C**) Representative images of H&E stained mouse dorsal skin from WT and slc15a4feeble mice demonstrated attenuated acanthosis in slc15a4feeble mice compared to WT mice on day 5 (n = 7, 5, 17, 15). Images taken at 200 × . Scale is the same in all pictures; scale bar = 100 µm. Data combined from 3 independent experiments. *p < 0.05, **p < 0.005, ***p < 0.0005, ****p < 0.0001 in unpaired two-sided parametric T tests.

**Figure 2:**
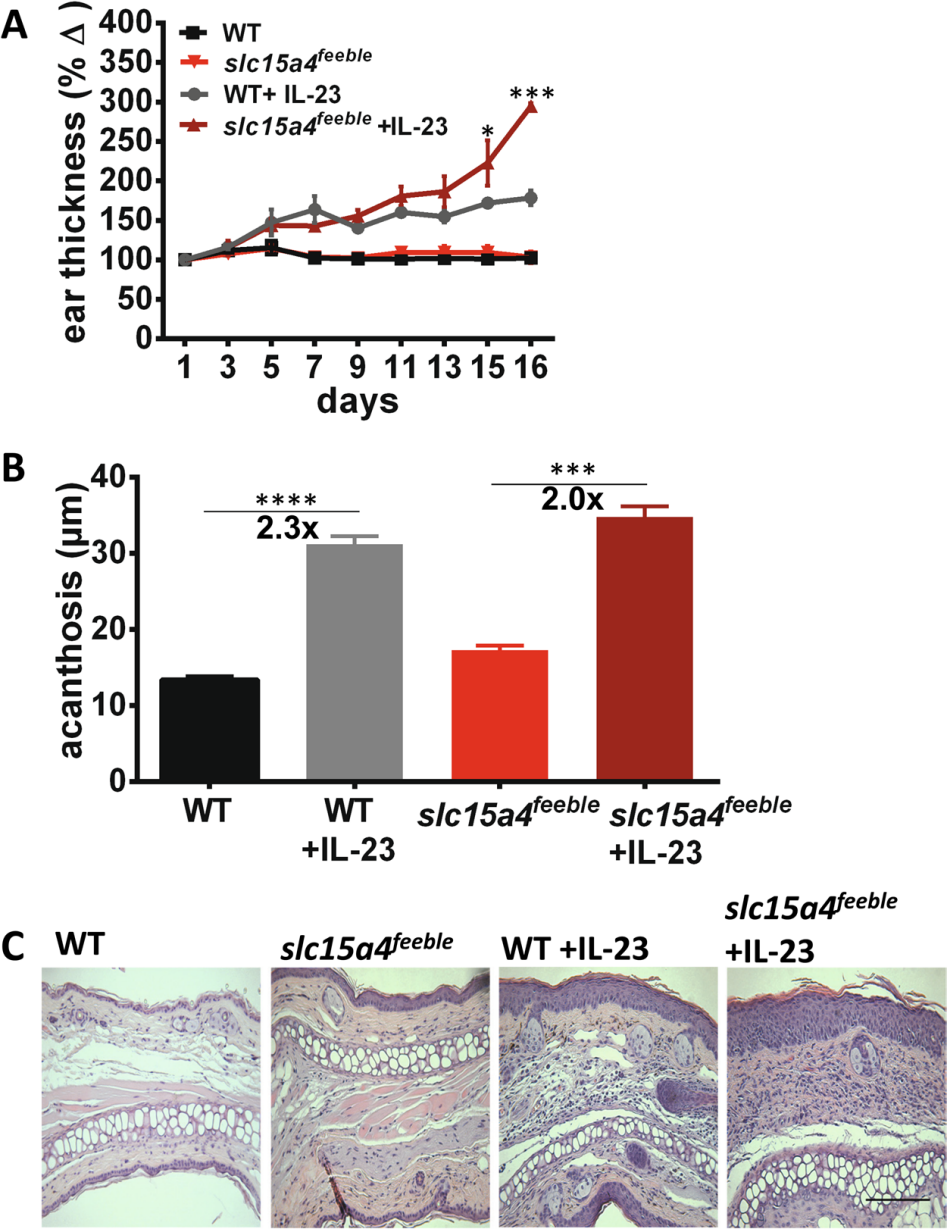
*s1c15a4*^*feeble*^ mice exhibit increased inflammation of the ear following intralesional injections of IL23. Ears of WT and *slc15a4*^*feeble*^ mice were injected every other day for 16 consecutive days with 20uL PBS containing 500 ng IL23. (**A**) On day 16, ears were collected for staining and measurement via H&E. (**B**) Ears were measured every other day with a micrometer, before injections (n = 7, 7, 6). (**C**) Representative H&E sections of mouse ears following treatment with IL23 (n = 7, 7, 6, 6). Images taken at 200x. Scale is the same in all pictures; scale bar = 100 µm. Data from two independent experiments each containing 6–7 mice/group. *p < 0.05, ***p < 0.0005, ****p < 0.0001 in unpaired two-sided parametric T tests.

We applied IMQ or vehicle control to the shaved backs of WT and *slc15a4*^*feeble*^ (*slc15a4* deficient mice) for 4 consecutive days and terminated the experiment on day 5 to be consistent with most IMQ studies^19^. Dramatically, *slc15a4*^*feeble*^ mice grossly appeared unaffected by IMQ, i.e. no hunching, withered appearance or decrease in movement was observed in contrast to their WT littermates. Consistently, IMQ-induced weight loss was not observed in *slc15a4*^*feeble*^ as compared to WT mice (Fig. 1A). Similarly, skin punch biopsies demonstrated attenuated acanthosis in *slc15a4*^*feeble*^ compared to WT mice as early as 24 hours post IMQ (Fig. 1B). This difference in skin thickening was greater by day 5 (Fig. 1B and C). To determine the specificity of *slc15a4* in this psoriasiform model, we executed a more chronic model of psoriasiform dermatitis that activates the downstream inflammatory cascade by direct intralesional injection of IL23 which promotes Th17 skewing. We found that after 11 days, *slc15a4*^*feeble*^ had increased ear skin thickness as compared to their WT littermates (Fig. 2A) and acanthosis similar to that induced by IMQ in WT mice (Fig. 2B). Thus, we reasoned that a *slc15a4* pro-inflammatory role was necessary in the IMQ model potentially by driving a very proximal event in the inflammatory pathway (i.e. pDC sensing of IMQ).

### *slc15a4* is required for IMQ-induced recruitment of CD4+ and SiglecH+ cells

**Figure 3:**
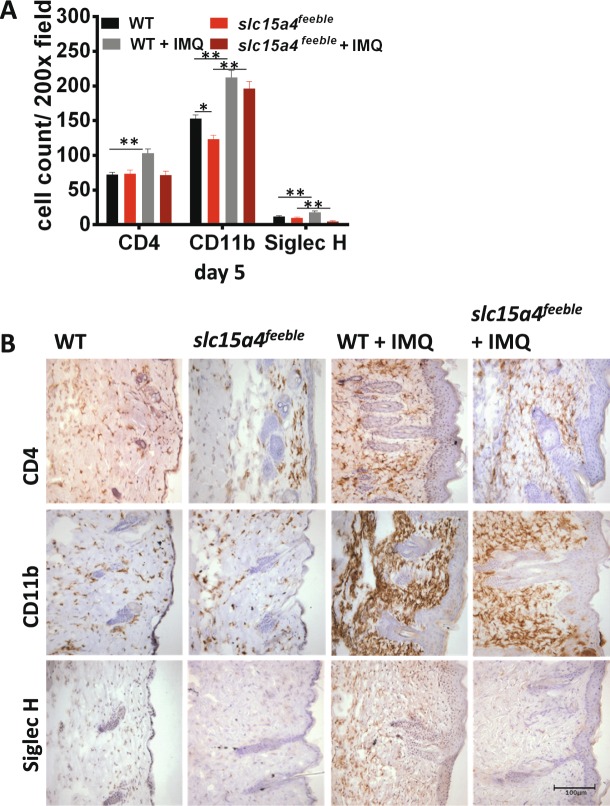
IMQ-dependent recruitment of CD4 + T cells into the dermis requires *slc15a4*. *slc15a4*^*feeble*^ and WT mice were shaved and treated with imiquimod (IMQ) or only shaved and euthanized after 5 days. (**A**) Quantitative measurements of CD4 T-cell (CD4), myeloid cell (CD11b) and pDC (Siglec H) infiltrate from immunostained dorsal mouse skin of WT and *slc15a4*^*feeble*^ mice 5 days post topical treatment with IMQ or control. n = 4, 5, 4, 5 mice for WT, *slc15a4*^*feeble*^, WT + IMQ, *slc15a4*^*feeble*^ + IMQ, respectively. (**B**) Representative immunostained sections of dorsal mouse skin at 6 days post treatment with IMQ. n = 4, 5, 4, 5 images from left to right. Images taken at 200x. Scale is the same in all pictures; scale bar = 100 µm. Data is from 1 of 3 similar experiments with 4–5 mice/group. *p < 0.05, **p < 0.005, in unpaired two-sided parametric T tests.

The IMQ model of psoriasiform dermatitis is driven by cytokine secreting CD4+ T cells of the lymphoid compartment in addition to CD11b+ myeloid cells^26,27^. These cell types are critical for the local and systemic effects of IMQ that we observed (Fig. 1). Thus, we compared the inflammatory infiltrate between treated and control WT and *slc15a4*^*feeble*^ mice on day 5 of IMQ treatment to determine if there was a difference in inflammatory cell infiltration by immunohistochemistry (IHC). We also quantitated the presence of the pDC marker SiglecH given the specific loss of function of pDC in *slc15a4* deficient mice. We observed an absence of CD4+ and SiglecH+ cells accumulating in the treated skin of *slc15a4*^*feeble*^ mice as compared to littermate controls by day 5 (Fig. 3A,B). These data implicated CD4+ T lymphocytes and pDC as potential cell types driving IMQ-driven weight loss and acanthosis via *slc15a4*. In contrast, there was an essentially normal accumulation of CD11b+ cells in *slc15a4*^*feeble*^ vs. WT mice. Albeit, *slc15a4*^*feeble*^ had a slightly lower absolute number of resident CD11b+ myeloid cells at baseline (Fig. 3A).

### *slc15a4* is required for IFN-I induction

**Figure 4:**
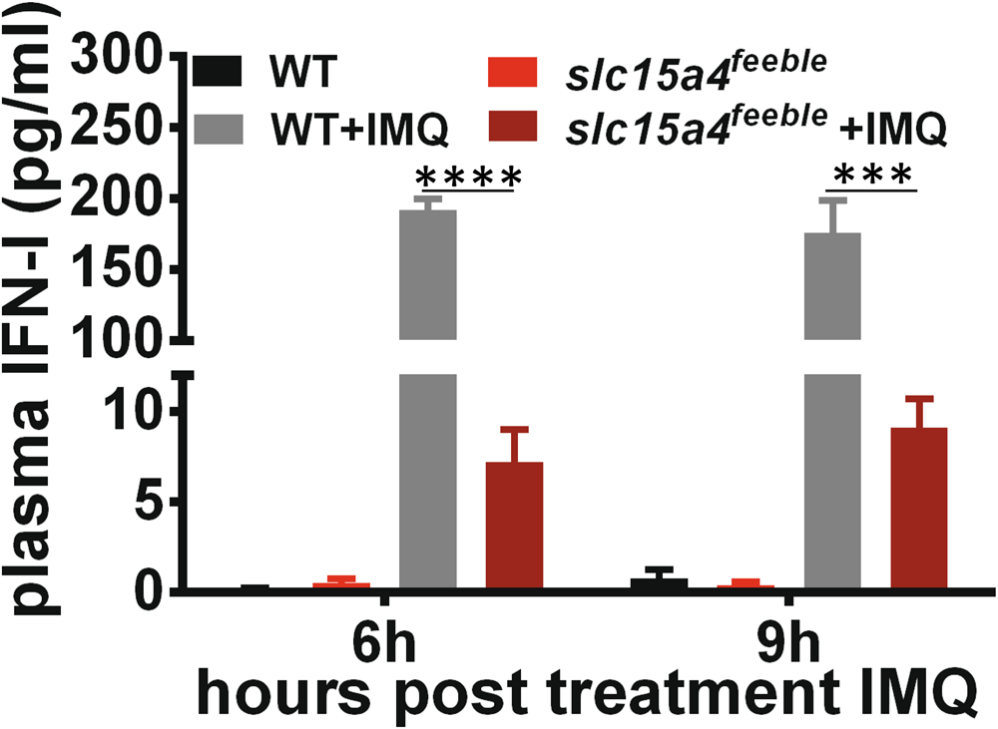
*slc15a4* is required for type 1 interferon induction. Plasma was collected 6 and 9 hours post IMQ from WT and *slc15a4*^*feeble*^ mice. IFN-I levels were than measured by ELISA. n = 4/group. 1 of 2 similar experiments shown. ***p < 0.0005, ****p < 0.0001 in unpaired two-sided parametric T tests.

pDC secrete ~1000x more IFN-I than any other cell type. Consistently, pDC are responsible for ~50% of all circulating IFN-I during acute viral infections despite being extremely rare cells^6^. Given the absence of SiglecH + pDC accumulating in *slc15a4*^*feeble*^, and the central role of pDC in IMQ responses^28–30^, we hypothesized that *slc15a4* would be required for IMQ-induced IFN-I. We found that the loss of *slc15a4* accounted for >1.5 log decrease in IFN-I production (Fig. 4).

### *slc15a4* contributes to systemic cytokine storm during topical IMQ treatment

**Figure 5:**
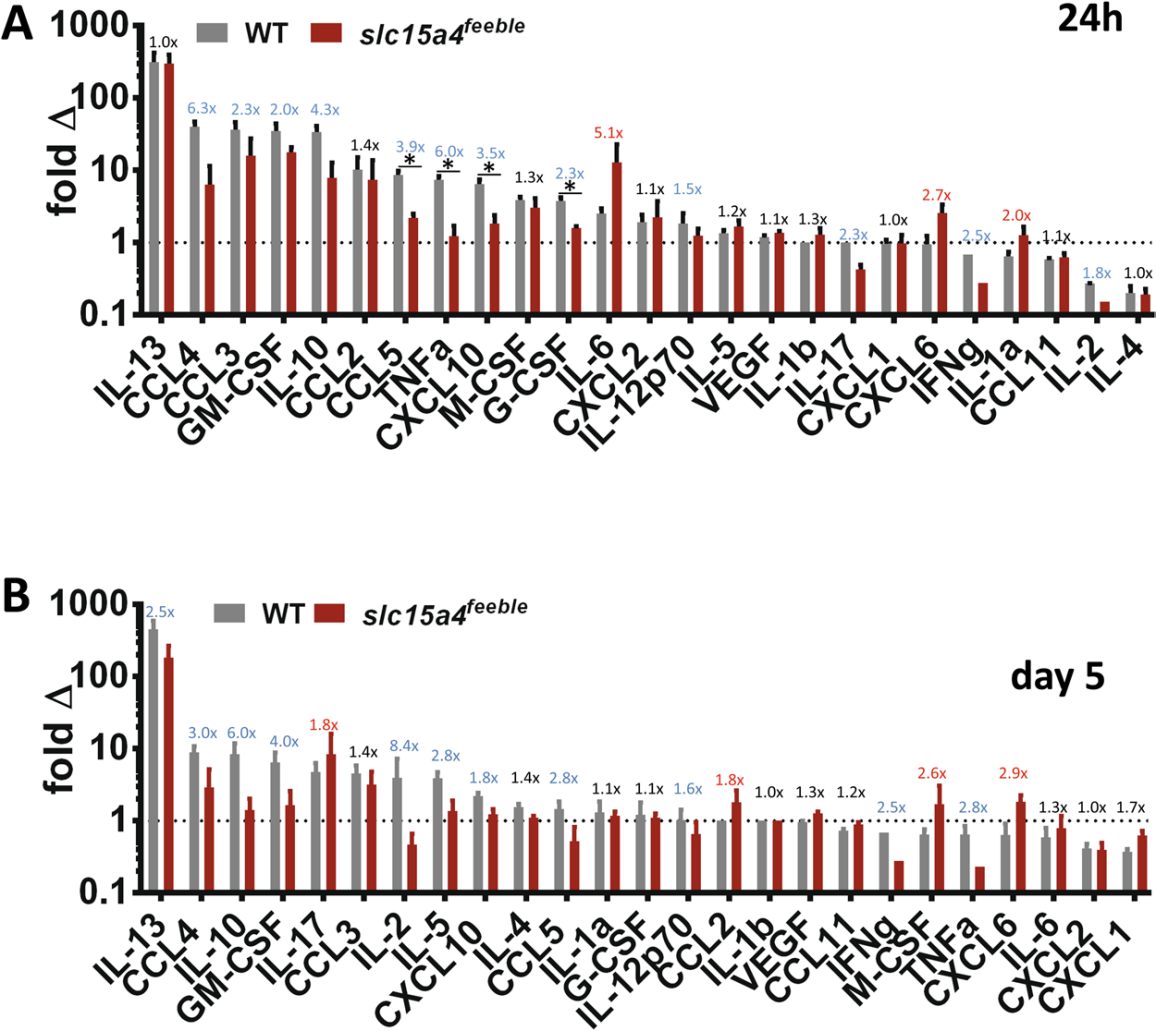
*slc15a4* contributes to IMQ driven cytokine storm. Inflammatory cytokine plasma concentrations were measured following IMQ treatment using a 25-cytokine luminex assay. Fold changes (IMQ-treated at the indicated timepoint/time = 0) are shown at 24 hours (**A**) and 5 days (**B**). Cytokines were rank-ordered for IMQ-induction in WT. The dotted line represents where IMQ treatment does not affect circulating cytokine levels. The ratio of IMQ-induced fold changes are displayed in blue (decreased), red (increased) and black (<1.5x changed) comparing mutant to WT. Data was combined from three independent experiments with a total of 5–13 mice per group. *p < 0.05 in unpaired T-test after multiple comparison correction.

IFN-I treatment is known to cause weight loss and general malaise that is associated with general inflammation^31^. Thus, we reasoned that IMQ-induced systemic IFN-I would likely propagate a broad induction of circulating inflammatory cytokines (“cytokine storm”). We measured 25 cytokines to address this possibility (Fig. 5). We found a potent induction of almost all 25 cytokines by IMQ. This induction was either unchanged or attenuated in *slc15a4*^*feeble*^. This attenuation was not generally due to a higher steady state concentration of cytokines in *slc15a4*^*feeble*^ as absolute plasma concentrations were overall very similar as compared to WT mice (Supp. Fig. 5). Only CXCL6(24 h and day 5), IL-1a (24 h only) and IL-6 (24 h only) were increased in *slc15a4*^*feeble*^ as compared to WT mice. These cytokines share a common nidus, inflammasome activation^32,33^. Induction of IL1b (the canonical output of the inflammasome) did not meet statistical significance, although it did trend in the same direction (Fig. 5).

### Unexpected more potent induction of trademark psoriasiform genes and inflammatory cytokines in skin of *slc15a4* deficient mice

**Figure 6:**
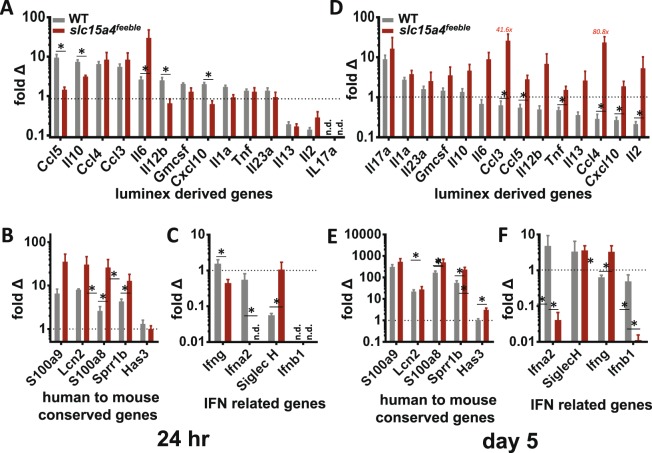
Unexpected more potent induction of trademark psoriasiform genes and inflammatory cytokines over time in skin of *slc15a4* deficient mice. RT-qPCR analysis of inflammatory genes elevated in 25plex cytokine analyses in IMQ-treated mice 24 hours (**A**) and 5 days (**D**) post treatment with IMQ. IL12b and IL23a were added to address Th1 and Th17 skewing, respectively. Cytokines were rank ordered from greatest to least induction in WT. Expression of trademark psoriasiform genes conserved between human and 5 mouse models is shown rank ordered for *human psoriasis* at 24 hours (**B**) and 5 days (**E**) post-treatment with IMQ. Otherwise, all other datasets are rank ordered for WT mouse induction. IFN-associated genes are shown 24 hours (**C**) and 5 days (**F**) post-treatment with IMQ. Fold change = IMQ relative quantity (RQ)/control RQ, see Materials and Methods for further details. Data is representative of 3 combined experiments with 5–13 mice/group. n.d. = not detected. *p < 0.05 in unpaired T-test after multiple comparison correction.

Lastly, we asked whether the IMQ-treated skin may contribute to the systemic increase in circulating cytokines. To address this we isolated total RNA from the treated skin 24 hours and on day 5 post initiation of treatment and quantitated gene expression for those cytokines identified in the luminex assay above, with validated TaqMan primer-probesets available. We added IL12b and IL23a given their central role in psoriasiform inflammation as the canonical gene products driving Th1 and Th17 skewing, respectively^34^. To further evaluate inflammasome associated genes, review of the literature demonstrated IL1f6 (IL-36a) and CXCL5 as additional IMQ-induced gene products^35,36^. RT-qPCR demonstrated induction of many of these cytokines at 24 h in WT mice (Fig. 6A) similar to what we observed in the plasma (Fig. 5A). However, this local skin induction was largely absent by day 5, consistent with downstream elaboration of circulating cytokines by immune cells which have left the treated skin in WT mice (Fig. 6A). This correlation with plasma proteins included the relative increase of inflammasome-related genes in in *slc15a4*^*feeble*^ compared to WT mice (Figs 5A and 6A) in addition to a moderate increase in the Th1 and Th17 skewing gene products IL12b and IL23a, respectively. Surprisingly, we found more potent gene induction of inflammatory cytokines within the skin of overall less-inflamed *slc15a4*^*feeble*^ mice by day 5 (Figs 5B and 6D). This could not be generally explained by lower basal gene expression in *slc15a4*^*feeble*^ mice (Fig. 6S). Taken together, we concluded that local skin gene induction of inflammatory cytokines may play a role in the first day of IMQ treatment. However, by day 5 the absence of weight loss and diminished cytokine storm in *slc15a4*^*feeble*^ could not be correlated with local gene expression. Thus, we reasoned that local gene expression could not serve as the predominant source for circulating cytokines on day 5. We than measured the most strongly increased trademark psoriasisform genes conserved across humans and 5 mouse models of psoriasiform dermatitis (including IMQ-induced)^35^ (Fig. 6B,E). Despite diminished IMQ-induced acanthosis in *slc15a4*^*feeble*^ (Fig. 1), we found more potent induction of the 5 most induced genes conserved across animal models and human psoriasis at 24 h and this increase was maintained on day 5 (Fig. 6B,E). We then confirmed that the genes whose regulation were most diminished in magnitude and over time in *slc15a4*^*feeble*^ were the type I interferon genes: Ifna2 and Ifnb1 but not type II interferon (gamma) (Fig. 6C,F). Thus, we concluded that increased induction of psoriasis-associated genes is not sufficient for maximal skin thickening and that diminished IFN-I, both local and systemic, was the most likely culprit.

## Discussion

The role of IFN-I in the IMQ model of psoriasiform dermatitis is controversial^5,20,22,23^. The most potent producer of IFN-I is the very rare pDC cell type. Thus efforts to target this cell type systemically for elimination can be difficult to access throughout the animal given the potential critical effects of even a few remaining cells. Additionally, compensatory responses are possible in the absence of this cell type. Therefore, we chose an alternative approach to access a potential therapeutic intervention for pDC-TLR7 driven immune pathology, genetic disruption of *slc15a4*. *Slc15a4* is required for pDC TLR7/9 sensing and subsequent TLR driven IFN-I production^7^. We and others previously found that this pathway was important in disease models^11,18,37^. Therefore, we hypothesized that studying *slc15a4* deficiency in the IMQ-induced psoriasiform dermatitis model may serve as an alternative strategy to specifically disrupt this pathway. These experiments also demonstrate the potential utility of targeting *slc15a4* therapeutically. *slc15a4*^*feeble*^ mice, lacking pDC sensing, exhibited attenuated local and systemic IMQ effects compared to WT mice.

It is also possible that the effect of *slc15a4* deficiency is mediated in part via B cells. However, this is less likely as the reported effects of *slc15a4* deficiency in B cells generally occur later than the phenotypes described here in an acute 5 day IMQ model. The only other cell type reported to be affected directly by *slc15a4* deficiency is mast cells. However, *slc15a4*^*−/−*^ mast cells degranulate more potently than their WT counterparts^38^ which would not explain our overall observations.

Given the acute effects of IMQ are largely driven via pDC TLR7 sensing^28–30^, we suspect that pDCs mediate the majority of the phenotype in *slc15a4*^*feeble*^. However, the opposite phenotype found in the IL23 model could involve B cells and/or mast cells. These cell types (especially mast cells in the acute setting) may participate in the apparently paradoxical observation of increased inflammasome-related systemic inflammatory cytokines (Fig. 5) and the unexpected increase in cutaneous pro-inflammatory gene expression (Fig. 6). Another explanation for these surprising findings could be inflammatory gene expression compensatory feedback in the absence of IFN-I, the latter of which is likely necessary for acanthosis as IFN-I protein and gene expression was consistently the most decreased pathway in *slc15a4*^*feeble*^ compared to WT mice.

Although *slc15a4* is tightly expressed in DC and B cells, we considered the possibility that *slc15a4*^*feeble*^ may have behavioral differences that contributed to our phenotypes^19^. However, no difference in grooming behaviors were observed between *slc15a4*^*feeble*^ and WT mice. Additionally, IMQ was applied until it was fully absorbed via visual inspection to help ensure that any inflammatory differences between WT and mutant mice were not confounded by differential uptake of IMQ or uptake via a different route (i.e. orally).

To understand the basis for the striking absence of weight loss in comparison to the more modest skin changes in *slc15a4*^*feeble*^, we examined circulating inflammatory cytokines as a possible explanation. We found diminished induction of 11/25 and 7/25 IMQ-induced circulating cytokines (24 h and day 5, respectively) that supported this hypothesis (Fig. 5). In addition, the most sustained inflammatory defect in *slc15a4*^*feeble*^ was an absence of IFN-I induction observed at all times at the level of protein and RNA expression when IFN-I is most prominently induced both systemically and locally (Figs 4 and 6). We suspect that the absence of weight loss in *slc15a4*^*feeble*^ during IMQ treatment is likely due to reduced IFN-I and/or other cytokines in circulation. Strikingly, three cytokines were upregulated at 24 h and 5 days post IMQ treatment in *slc15a4*^*feeble*^ versus WT mice. These were all inflammasome-related cytokines^32,33,39,40^. The IMQ psoriasiform model is mediated by the TLR7 vs. inflammasome pathways^20^. *slc15a4* is required for TLR7 sensing via pDC consistent with diminished acanthosis, weight loss (Fig. 1), immune cell recruitment (Fig. 3), cytokine storm (Figs 4 and 5) and cutaneous induction of several genes at 24 h (Fig. 6). However, some inflammation remained in *slc15a4* during IMQ topical treatment as measured by acanthosis (Fig. 1) with preserved recruitment of myeloid immune cells (Fig. 3). Our data supports the activation of the inflammasome as a potential etiology for this residual acanthosis whose activation is independent of *slc15a4* consistent with its reported function. Specifically, the relatively heightened concentration of inflammasome-related cytokines in the plasma (Fig. 5) and inflammasome-related genes in the treated skin (Fig. 6) support a molecular basis for residual IMQ-induced inflammation without pDC-TLR7 sensing. Residual markers of molecular inflammation in *slc15a4*^*feeble*^ was most striking for the trademark genes whose induction is conserved across human psoriasis and mouse models^35^ at 24 h and subsequently, most inflammatory cytokines surveyed on day 5 (Fig. 6). Taken together, we reason that pDC-TLR7 sensing can be targeted via *slc15a4* and is critical for the inflammatory cascade which results in significant morbidity and weight loss in the IMQ model of psoriasisform dermatitis. In this IMQ-induced model, loss of *slc15a4* is most tightly tied to a defect in IFN-I production while an inflammasome signature is preserved.

## Materials and Methods

### Mice

All mice used in this study were housed in the Case Western Reserve University Biomedical Research Building Animal Facility. C57BL/6 J mice were bred locally or ordered from Jackson Laboratories. *slc15a4*^*feeble/feeble*^ (MGI: 4835997) mutants have been described previously^7^.

### Imiquimod challenge model

Briefly, *slc15a4*^*feeble*^ and C57BL/6 J mice were used at 6–8 wks of age and gender did not determine different outcomes. Mouse dorsal skin was treated with IMQ, for 1 day or 5 consecutive days, followed by harvest of tissue and plasma. The dorsal skin of wildtype and *feeble* mice were shaved prior to IMQ application. 0.05 g/mouse of IMQ cream (5%), vehicle only, or no cream was applied to the shaved skin of the mice. Clinical phenotypes (weight loss and acanthosis as described previously^19^) were identical between vehicle only and no cream conditions.

### IL23 challenge model

Briefly, *slc15a4*^*feeble*^ and C57BL/6 J mice were used at 6–8 wks of age and gender did not affect different outcomes. Mice received intralesional injections of 20 μl PBS, either alone or containing 500 ng recombinant mouse IL-23 (eBioscience), using a 26-gauge needle every other day for 16 days as previously described^41^. Ear thickness was measured via micrometer before injection on day 0 and thereafter on days without injections.

### Immunohistochemistry

Skin was embedded fresh in Tissue Freezing Media (TFM; Triangle Biomedical), flash frozen in liquid nitrogen and immediately stored at −80 °C. Remaining treated dorsal skin was flash frozen and stored at −80 °C for use in RNA experiments. Immunohistochemistry was performed on 8 μm sections. Antibodies and isotype controls were detected using either rabbit anti-rat IgG biotinylated or goat anti-rabbit IgG biotinylated, amplified with Avidin/Biotinylated Enzyme Complex (ABC, Vector) and were visualized using the enzyme substrate diaminobenzidine (Vector). Slides were counterstained with hematoxylin and imaged as previously described^42^. Three different observers have quantified each image manually on two occasions.

### RNA Isolation and RT-qPCR

Dorsal skin was homogenized in TRIzol using a Mikro-Dismembrator (Sartorius Stedim Biotech, Edgewood, NY) and RNA isolated using RNeasy Mini Kit (Qiagen, Valencia, CA). Transcriptor First Strand cDNA Synthesis Kit (Roche) was performed following the manufacturer’s instructions. RT-qPCR was than performed using TaqMan microfluidic array technology from Applied Biosystems on an ABI 7900HT. Relative Quantity (RQ) expression levels were calculated using the comparative CT method (ΔΔCT) and normalized by GAPDH for: Ifn-gamma, Ifn-beta, Siglec H, Gmcsf, Il-2, Il-7, Il-10, Ccl5, Ccl3, Ccl4, Il-13, Il-23, S100a7a, S100a9, S100a8, Cxcl1010, IL-1f6, Tnf-a, Il-12p40, Serpinb3c, IL-1 alpha, Has3, Lcn2, Sprr1b, Pcdh21, Icos, Il-17, Ccl5, Ifna2, and Lor. Absolute values of all measured genes, at baseline and induction, is described in the Supplementary Figures 1, 2.

### Analysis of circulating cytokines

Plasma collected at 24 h and 5 days was interrogated with a 25plex luminex screening assay (R&D systems) on a Biorad Bioplex Magpix Multiplex Luminex Microplate Reader by the CWRU Bioanalyte core for IL-12p70, VEGF, IL-1beta, CCL2, CXCL2, IL-6, IL-4, IL-2, TNFalpha, CXCL1, IL-17, IL-10, IL-13, IL-5, IFNgamma, CXCL10, CCL5, G-CSF, M-CSF, CCL4, IL-1alpha, CCL3, GM-CSF, CXCL6, and CCL11. IFN-I was measured in plasma by ELISA per manufacturer’s instructions (PBL Assay Science). Absolute values of all measured circulating cytokines, at baseline and induction, is described in the Supplementary Figures 3–8.

### Statistical Analysis

Unpaired two-sided parametric T-tests were performed between WT and WT + IMQ groups at each time point in addition to comparing *slc15a4*^*feeble*^ and *slc15a4*^*feeble*^ + IMQ groups where indicated. When more than 4 groups were compared, statistical significance was determined using the Holm-Sidak method to correct for multiple comparisons with alpha = 5%, as indicated. Each row was analyzed individually, without assuming a consistent SD.

### Ethical Approval

Experimental protocols were approved by Case Western Reserve University Institutional Animal Care and Use Committee (IACUC) institutional committee under IACUC protocol # 2014-0106. Specifically, Less than 5 mice were housed per cage with daily monitoring to ensure optimal animal welfare and to ameliorate suffering. All mouse studies have been conducted according to national and international guidelines and in accordance with institutional regulations governing animal care and useas detailed in IACUC# 2014-0106.

## Electronic supplementary material

Supplementary Dataset

## Data Availability

The datasets generated during and/or analysed during the current study are available from the corresponding author on reasonable request.
