# Supplementary material for: A requirement for slc15a4 in imiquimod-induced systemic inflammation and psoriasiform inflammation in mice
Source: Sci Rep. 2018 Sep 27;8:14451. doi: 10.1038/s41598-018-32668-9 (PMC6160456; doi:10.1038/s41598-018-32668-9)
Supplement: Supplementary file 1 — Supplementary Dataset [file 41598_2018_32668_MOESM1_ESM.pdf]

A requirement for *slc15a4* in imiquimod-induced systemic inflammation and psoriasiform inflammation in mice

Alexis D. Griffith[1], Asifa K. Zaidi[1], Ashley Pietro[1], Matthew Hadiono[1], Jessica Yang[1], Rachel Davis[1], Daniel L. Popkin MD PhD[1,2]*

[1] Department of Dermatology, Case Western Reserve University Hospitals, Cleveland, OH 44106

[2] Departments of Dermatology, Pathology, Molecular Biology and Microbiology. Case Western Reserve University Hospitals, Cleveland, OH 44106

Corresponding author: Correspondence should be addressed to D.L.P.

*****daniel.popkin@case.edu

Supplementary Information File

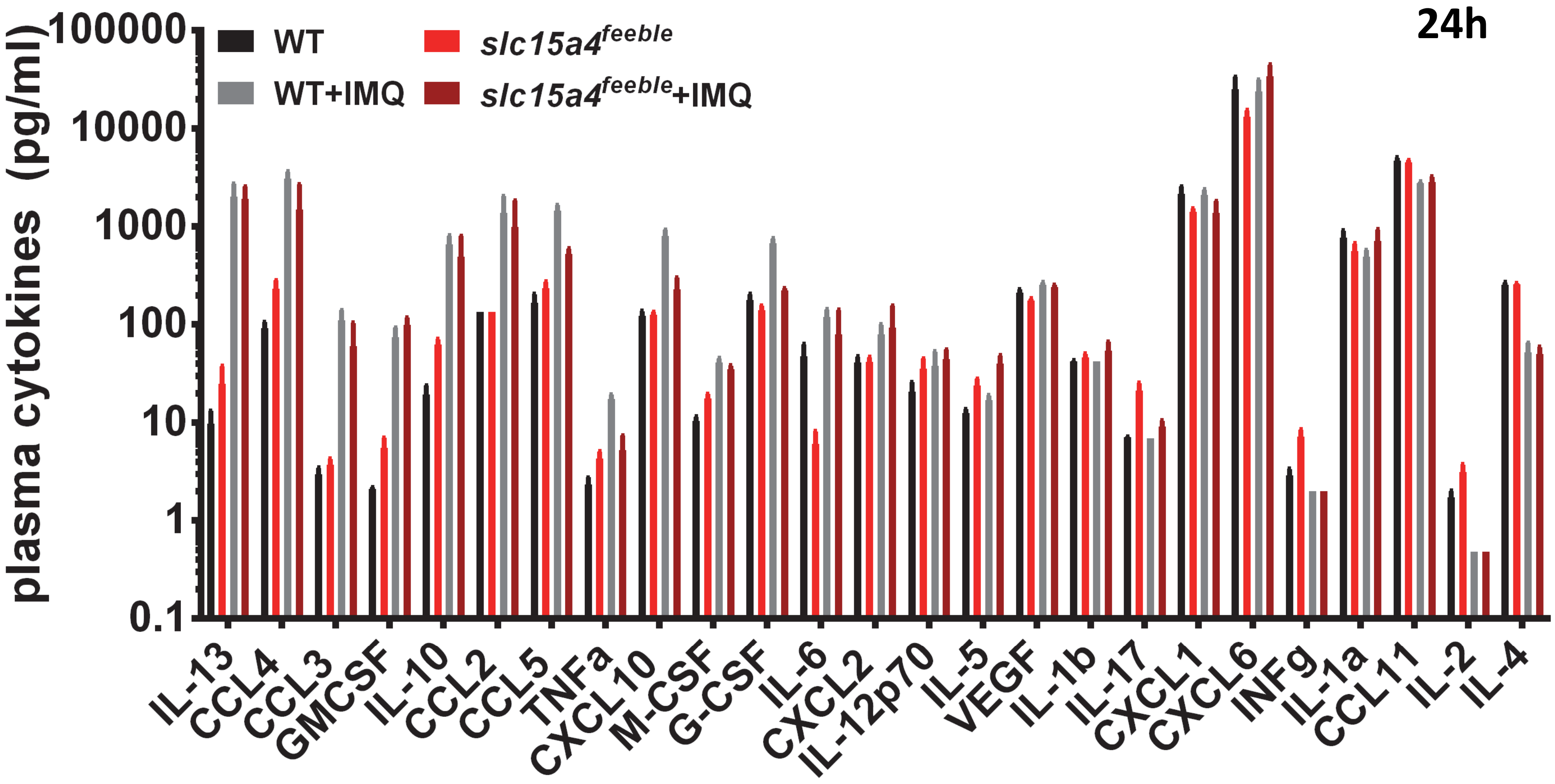

24h
WT
WT+IMQ
slc15a4feeble
slc15a4feeble+IMQ
plasma cytokines (pg/ml)
100000
10000
1000
100
10
1
0.1
IL-13
CCL4
CCL3
GMCSF
IL-10
CCL2
CCL5
TNFa
CXCL10
M-CSF
G-CSF
IL-6
CXCL2
IL-12p70
IL-5
VEGF
IL-1b
IL-17
CXCL1
CXCL6
INFg
IL-1a
CCL11
IL-2
IL-4

Supp. Fig. 1

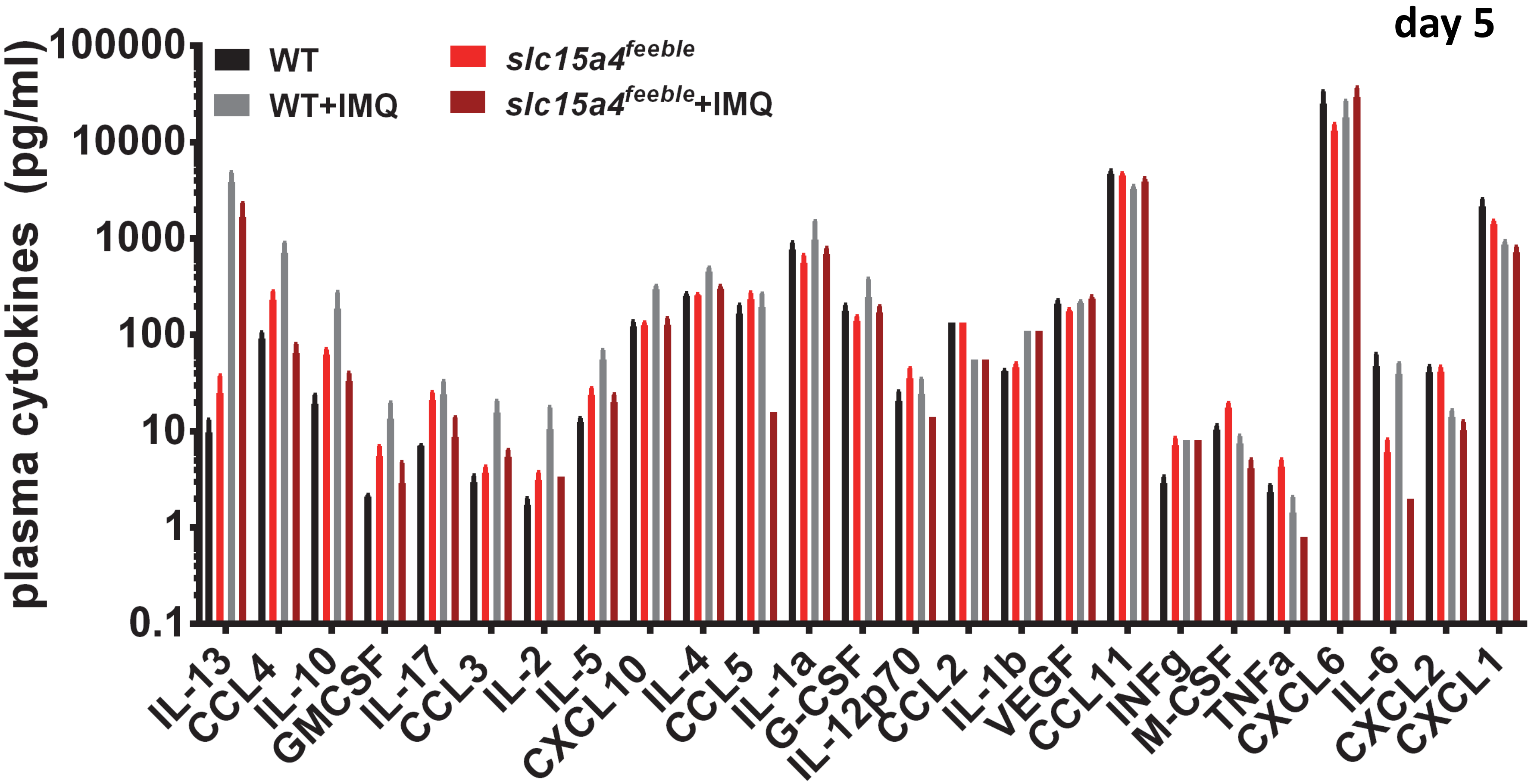
day 5
plasma cytokines (pg/ml)
100000
10000
1000
100
10
1
0.1
WT
WT+IMQ
slc15a4feeble
slc15a4feeble+IMQ
IL-13
CCL4
IL-10
GMCSF
IL-17
CCL3
IL-2
IL-5
CXCL10
IL-4
CCL5
IL-1a
G-CSF
IL-12p70
CCL2
IL-1b
VEGF
CCL11
INFg
M-CSF
TNFa
CXCL6
IL-6
CXCL2
CXCL1

Supp. Fig. 2

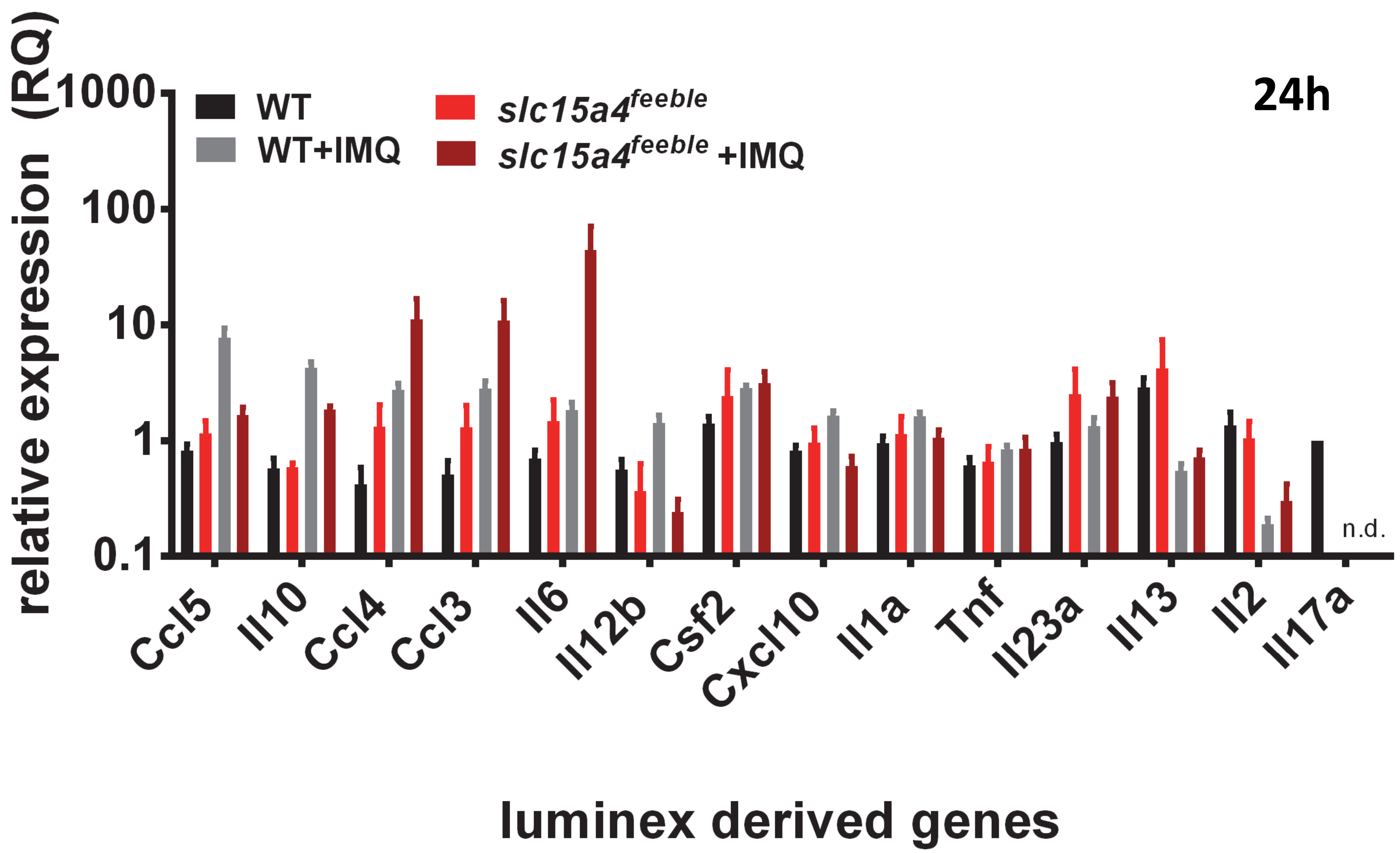

24h
WT
WT+IMQ
slc15a4feeble
slc15a4feeble +IMQ
relative expression (RQ)
1000
100
10
1
0.1
n.d.
Ccl5
Il10
Ccl4
Ccl3
Il6
Il12b
Csf2
Cxcl10
Il1a
Tnf
Il23a
Il13
Il2
Il17a
luminex derived genes

Supp. Fig. 3

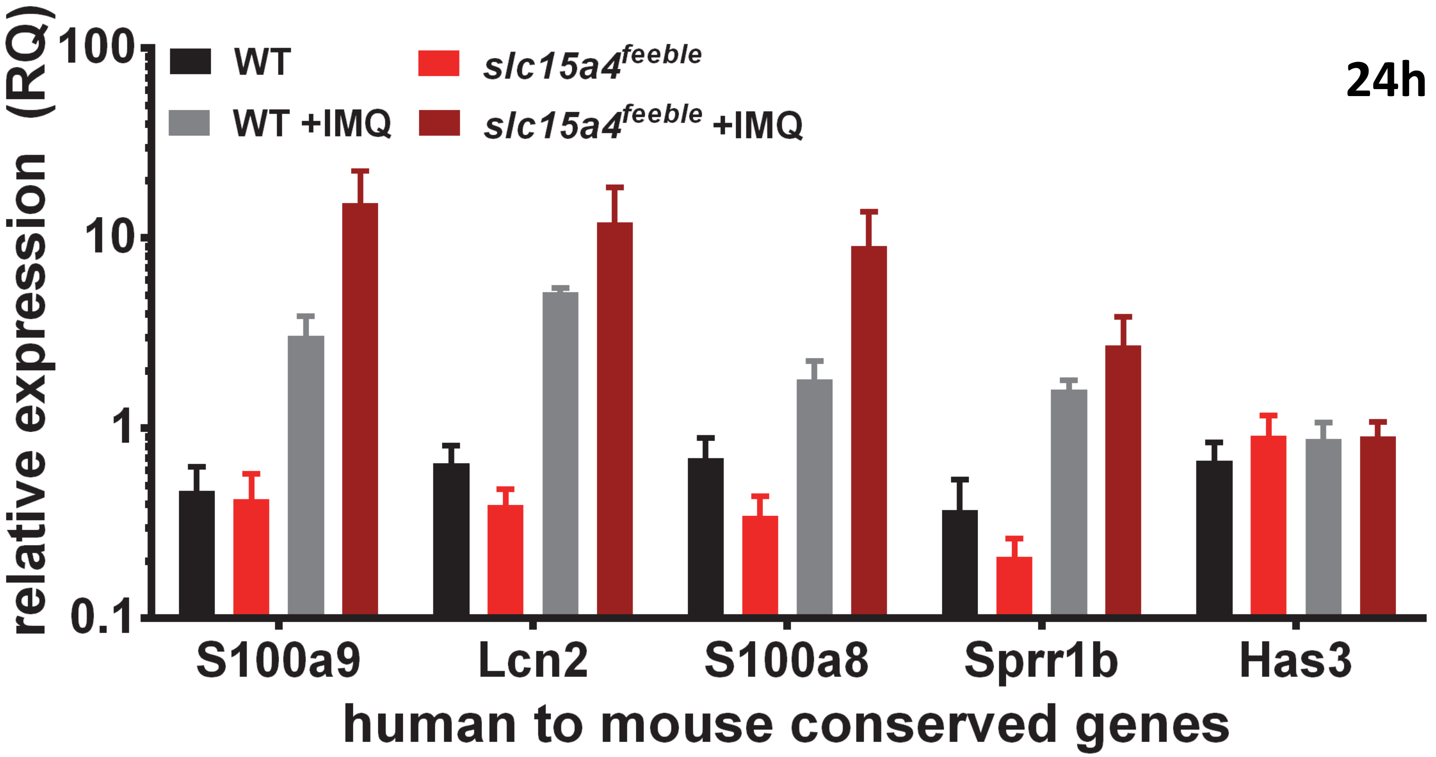

relative expression (RQ)
100
10
1
0.1
WT
WT +IMQ
slc15a4feeble
slc15a4feeble +IMQ
24h
S100a9
Lcn2
S100a8
Sprr1b
Has3
human to mouse conserved genes

Supp. Fig. 4

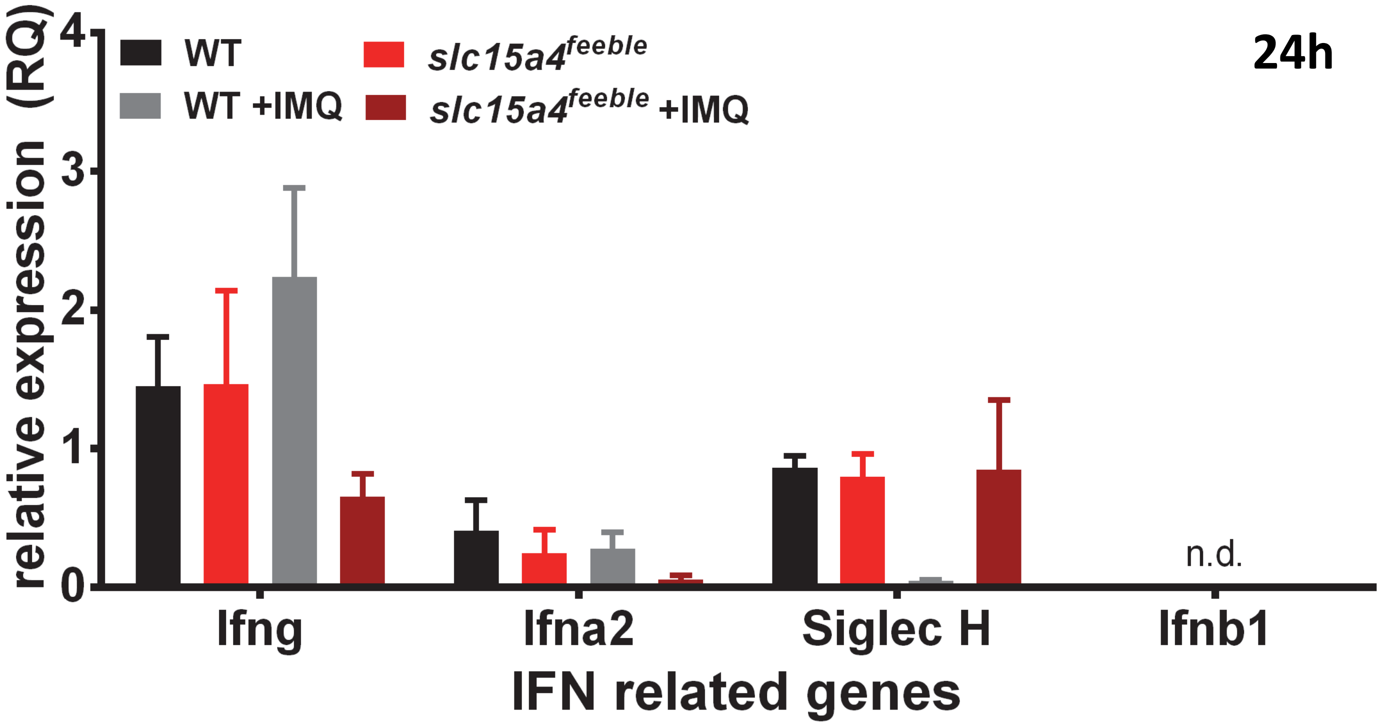

relative expression (RQ)
4
3
2
1
0
WT
WT +IMQ
slc15a4feeble
slc15a4feeble +IMQ
24h
n.d.
Ifng
Ifna2
Siglec H
Ifnb1
IFN related genes

Supp. Fig. 5

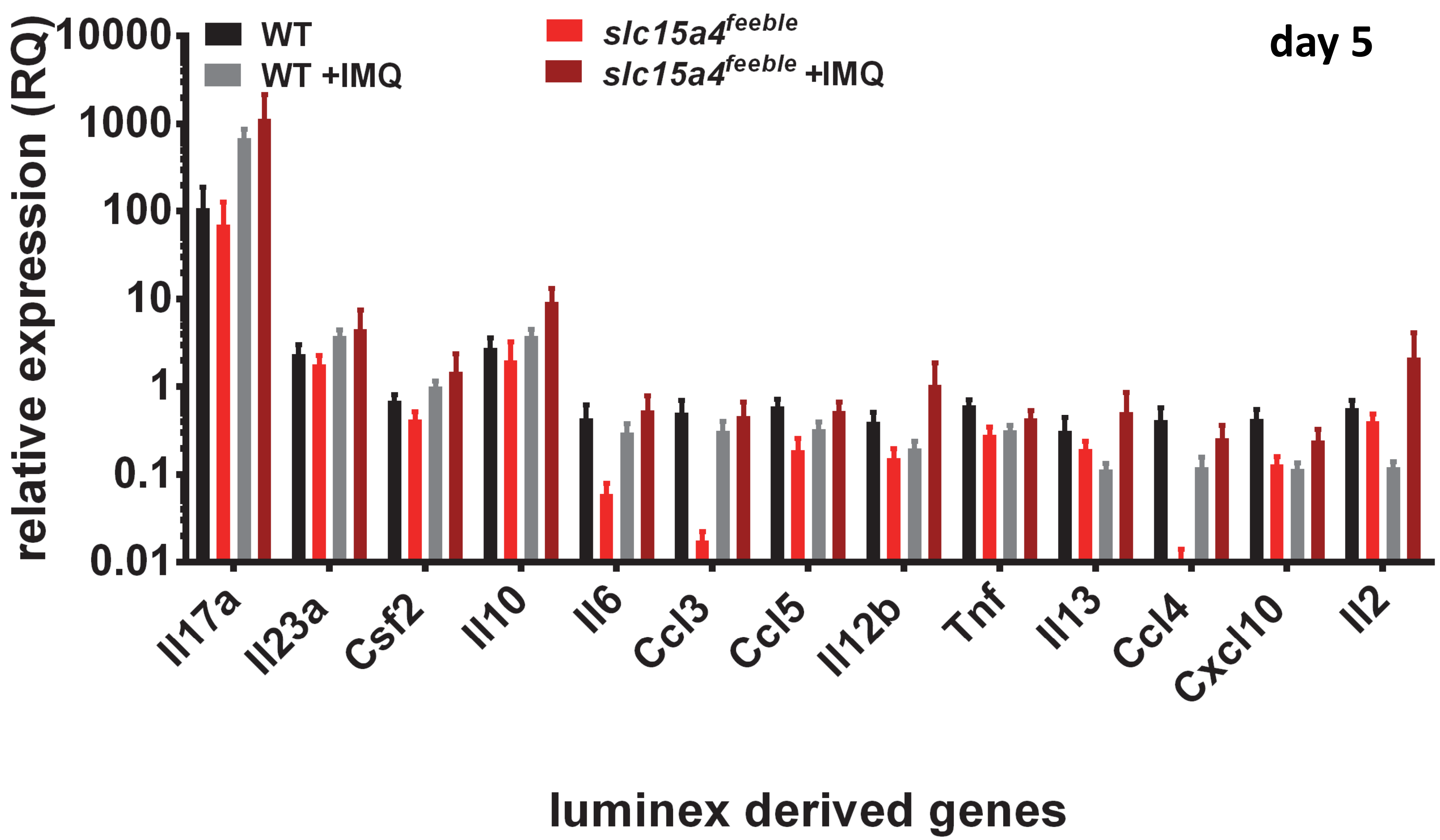
relative expression (RQ)
10000
1000
100
10
1
0.1
0.01
WT
WT +IMQ
slc15a4feeble
slc15a4feeble +IMQ
day 5
Il17a
Il23a
Csf2
Il10
Il6
Ccl3
Ccl5
Il12b
Tnf
Il13
Ccl4
Cxcl10
Il2
luminex derived genes

Supp. Fig. 6

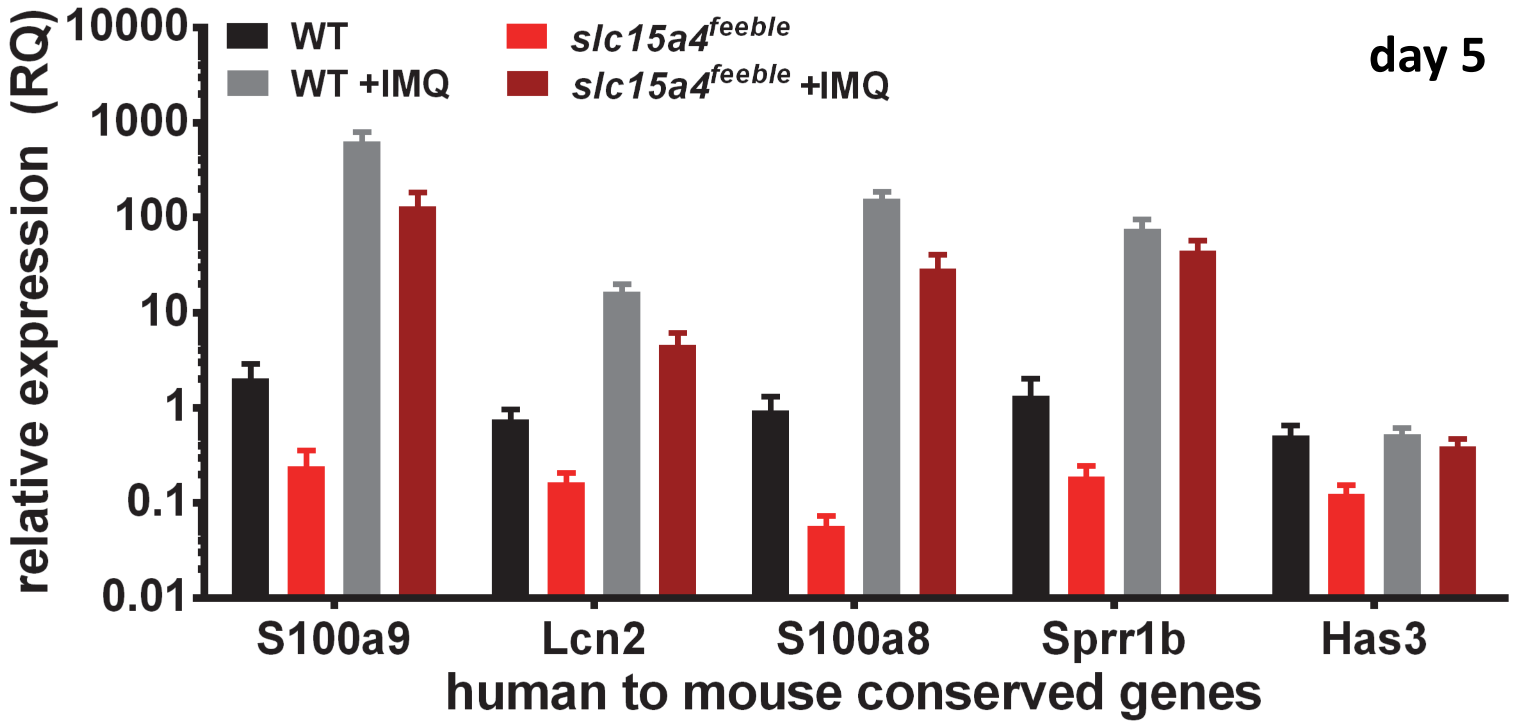

relative expression (RQ)
10000
1000
100
10
1
0.1
0.01
WT
WT +IMQ
slc15a4feeble
slc15a4feeble +IMQ
day 5
S100a9
Lcn2
S100a8
Sprr1b
Has3
human to mouse conserved genes

Supp. Fig. 7

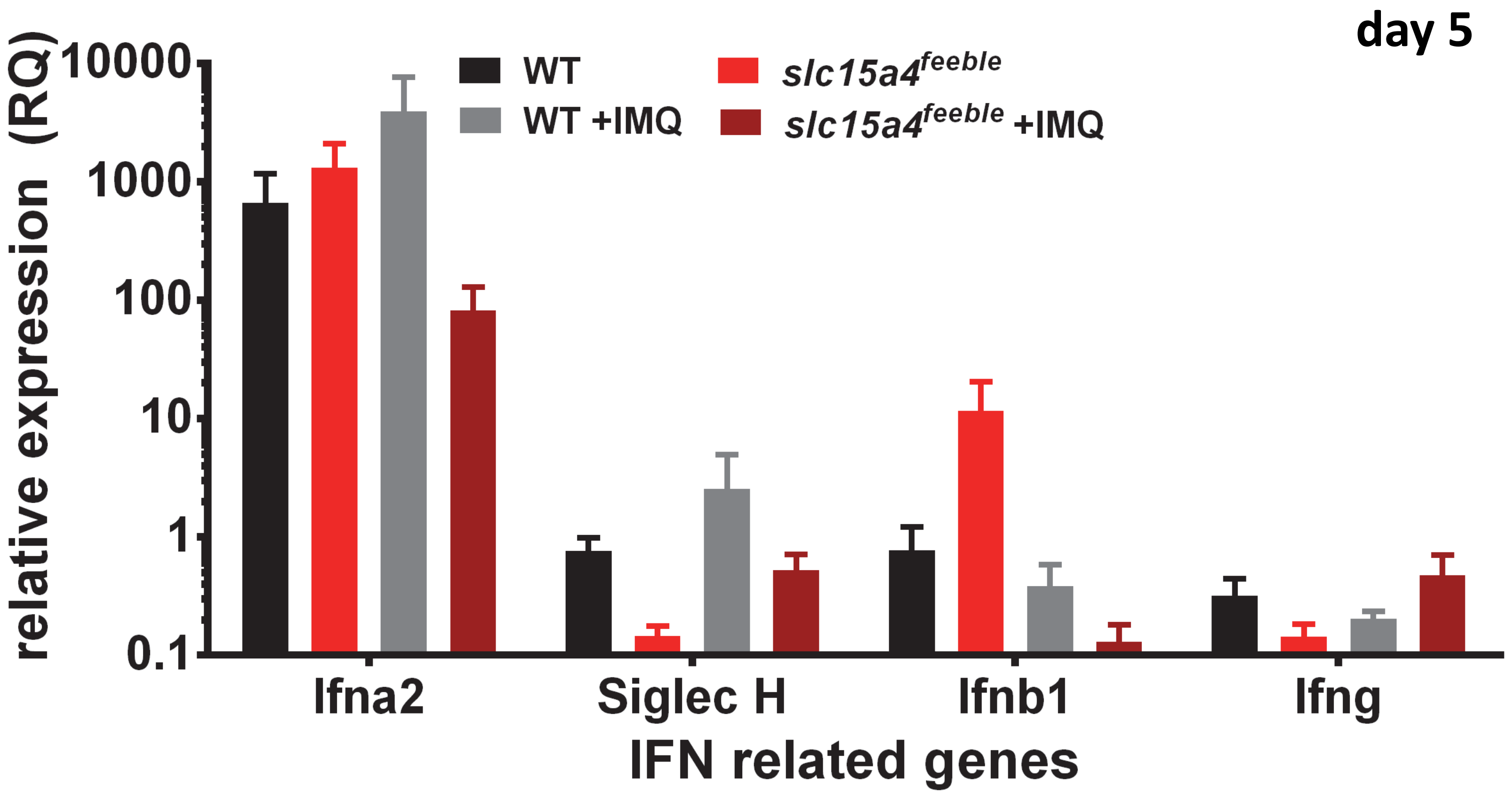

day 5
relative expression (RQ)
10000
1000
100
10
1
0.1
WT
WT +IMQ
slc15a4feeble
slc15a4feeble +IMQ
Ifna2
Siglec H
Ifnb1
Ifng
IFN related genes

Supp. Fig. 8

Supplemental Figure 1: Absolute values (pg/ml) of IMQ-drive cytokines in WT and *slc15a4*^{feeble} mice at 24 hours. Inflammatory cytokine expression levels were measured from mouse plasma collected following no treatment vs. IMQ treatment using a 25 cytokine luminex screening assay. IMQ divided by baseline cytokine levels (IMQ-induction) is shown after 24 hours. Data is representative of 3 combined experiments with a total of 5-13 mice per group. *= $p<0.05$ in paired T-test.

Supplemental Figure 2: Absolute values (pg/ml) of IMQ-driven cytokines in WT and *slc15a4*^{feeble} mice at 5 days. Inflammatory cytokine expression levels were measured from mouse plasma collected following no treatment vs. IMQ treatment using a 25 cytokine luminex screening assay. IMQ divided by baseline cytokine levels (IMQ-induction) is shown 5 days post continuous IMQ treatment. Data is representative of 3 combined experiments with a total of 5-13 mice per group. *= $p<0.05$ in paired T-test.

Supplemental Figure 3: Relative expression of inflammatory genes via RT-qPCR in WT and *slc15a4*^{feeble} mice at 24 hours. Gene expression was measured via RT-qPCR ThermoFisher 384 well TaqMan gene expression cards on RNA isolated from the dorsal skin of WT and *slc15a4*^{feeble} mice treated with IMQ for 24 hours. Cytokines were limited to those for which validated TaqMan primer-probe sets were available. Cytokines are shown rank ordered from greatest to least induction in WT. Expression was measured via RT-qPCR ThermoFisher 384 well TaqMan gene expression cards. * = $p<0.05$ in paired T-test. Data is representative of 3 combined experiments with 5-13 mice/ group.

Supplemental Figure 4: Relative expression of the 5 most highly conserved genes across human and mouse models of psoriasis via RT-qPCR in WT and *slc15a4*^{feeble} mice at 24 hours. Gene expression was measured via RT-qPCR ThermoFisher 384 well TaqMan gene expression cards on RNA isolated from the dorsal skin of WT and *slc15a4*^{feeble} mice treated with IMQ for 24 hours. Genes are shown rank ordered from greatest to least induction in human models of psoriasis. * = p<0.05 in paired T-test. Data is representative of 3 combined experiments with 5-13 mice/ group.

Supplemental Figure 5: Relative expression of the IFN pathway related genes via RT-qPCR in WT and *slc15a4*^{feeble} mice at 24 hours. Gene expression was measured via RT-qPCR ThermoFisher 384 well TaqMan gene expression cards on RNA isolated from the dorsal skin of WT and *slc15a4*^{feeble} mice treated with IMQ for 24 hours. Genes are shown rank ordered from greatest to least induction in WT. * = p<0.05 in paired T-test. Data is representative of 3 combined experiments with 5-13 mice/ group.

Supplemental Figure 6: Relative expression of inflammatory genes via RT-qPCR in WT and *slc15a4*^{feeble} mice at 5 days. Gene expression was measured via RT-qPCR ThermoFisher 384 well TaqMan gene expression cards on RNA isolated from the dorsal skin of WT and s*lc15a4*^{feeble} mice treated with IMQ for 5 consecutive days. Cytokines were limited to those for which validated TaqMan primer-probe sets were available. Cytokines are shown rank ordered from greatest to least induction in WT. Expression was measured via RT-qPCR ThermoFisher 384 well TaqMan gene expression cards. * = p<0.05 in paired T-test. Data is representative of 3 combined experiments with 5-13 mice/ group.

Supplemental Figure 7: Relative expression of the 5 most highly conserved genes across human and mouse models of psoriasis via RT-qPCR in WT and *slc15a4*^{feeble} mice at 5 days. Gene expression was measured via RT-qPCR ThermoFisher 384 well TaqMan gene expression cards on RNA isolated from the dorsal skin of WT and *slc15a4*^{feeble} mice treated with IMQ for 5 consecutive days. Genes are shown rank ordered from greatest to least induction in human models of psoriasis. * = $p<0.05$ in paired T-test. Data is representative of 3 combined experiments with 5-13 mice/ group.

Supplemental Figure 8: Relative expression of IFN pathway related genes via RT-qPCR in WT and *slc15a4*^{feeble} mice at 5 days. Gene expression was measured via RT-qPCR ThermoFisher 384 well TaqMan gene expression cards on RNA isolated from the dorsal skin of WT and *slc15a4*^{feeble} mice treated with IMQ for 5 consecutive days. Genes are shown rank ordered from greatest to least induction in WT. * = $p<0.05$ in paired T-test. Data is representative of 3 combined experiments with 5-13 mice/ group.
